# The physical, psychological, and social impacts of participation in the Invictus Pathways Program: A qualitative analysis of veterans’ perceptions and experiences

**DOI:** 10.1371/journal.pone.0287228

**Published:** 2023-10-30

**Authors:** Dannielle Post, Amy Baker, Steven Milanese, Suzana Freegard, Gaynor Parfitt

**Affiliations:** 1 Alliance for Research in Exercise, Nutrition, and Activity (ARENA), Allied Health and Human Performance, University of South Australia, Adelaide, South Australia, Australia; 2 Allied Health and Human Performance, University of South Australia, Adelaide, South Australia, Australia; 3 International Centre for Allied Health Evidence (ICAHE), Allied Health and Human Performance, University of South Australia, Adelaide, South Australia, Australia; George Mason University, UNITED STATES

## Abstract

**Introduction:**

UniSA’s Invictus Pathways Program (IPP) is motivated by the spirit of the Invictus Games to mobilise the benefits of sport to aid physical, psychological, and social wellbeing. Originally developed to assist veterans to train for and participate in the Invictus Games, the program has extended its scope to promote recovery and wellbeing for all veterans through physical activity. This paper describes the expectations and experiences of the IPP from the perspective of program participants.

**Methods:**

Objective measures of physical and psychological wellbeing were collected by survey, to enable description of the participating veterans’ wellbeing status. Semi-structured interviews were conducted with 15 participants of the IPP who had not participated in an Invictus Games or Warrior Games. Reflexive thematic analysis was used to analyse the interview data. Coding and themes were developed through a mixture of inductive and deductive approaches to analysis. Initial themes related to previous life experience, expectations of participation, and outcomes of participation were preconceived, but the analysis provided scope for an inductive approach to formulate additional themes.

**Findings:**

Five of the participants had very high K10 scores, and scores above the norm for PCL-C, whilst one would be classified with an alcohol disorder. The qualitative analysis identified five higher order themes: Life experiences prior to participation in the IPP, Making a choice to participate in the IPP, Expectations of participation in the IPP, Impact of participation in the IPP, and Future Plans. There were perceptions that the IPP was beneficial for the participating veterans, irrespective of their physical and psychological health status. Participants described the positive impact of the IPP on their physical fitness, their social engagement, and their sense of belonging within the IPP and the university. Participants perceived the IPP to be an opportunity for them to ‘give back’ by contributing to the education of the students delivering the IPP. Participants reported the intention to continue being physically active. For some, this meant selection in an Invictus Games team, for others, this meant getting involved in community sporting organisations.

**Conclusion:**

The Invictus Pathways Program has been shown to have a positive impact on the physical and psychological wellbeing of the veterans who participated in its initial stages. As the program evolves, the longitudinal impact of participation, for veterans and their families, will be assessed.

## Introduction

It is estimated that over 581,000 Australians have served or are currently serving in the Australian Defence Force (ADF) [1], many of whom will be physically or psychologically affected by their military engagement. Over the years, the number of casualties in combat has decreased while the number of returned injured military personnel has increased, most likely due to improvements in medical knowledge, military responses in the field, and protective body gear [2]. The increasing number of injured returned military personnel has led to increasing demands on rehabilitation services.

While many injuries can be successfully treated, some health conditions and injuries are too debilitating for military personnel to continue with their service, and they are forced to leave the service. Approximately 5000 military personnel transition out of the ADF each year with just over one fifth of those being discharged on medical grounds [3]. The transition process can be a traumatic experience in itself, with veterans reporting difficulty reintegrating into society, loss of identity, issues with gaining employment, financial concerns, and for some, finding purpose in life [4–8]. As of 2022, 13 percent of former serving members continue to require assistance with daily activities including mobility, self-care, and communication [9].

Mental health conditions, specifically Post-Traumatic Stress Disorder (PTSD), depressive disorders, and anxiety disorders, as well as alcohol dependence and abuse are the most common health conditions affecting veterans [10]. Findings of the Australian Mental Health and Wellbeing Transition Study suggest that 46% of transitioned full-time ADF members, who had transitioned in the previous five years, have experienced a mental health disorder in the past 12 months [3]. These figures are even higher for those who have been discharged on medical grounds. At the population level, one-in-five Australians (21.4% or 4.2 million people) reported having a mental disorder in the 12 months prior [11]. For males, mental health conditions were reported by 22 percent who had served compared to 18 percent who had not served. Data for females for the same period of time were not available, attributed to the small proportion who had ever served (14 percent). As of 2019, rates of suicide are 24 percent higher for ex-serving males and twice as high for ex-serving females compared to the general population [12].

Developed in 2017, through a collaboration between the University of South Australia (UniSA) and The Road Home (now Military and Emergency Services Health Australia: MESHA), UniSA’s Invictus Pathways Program (IPP) is motivated by the spirit of the Invictus Games to mobilise the benefits of physical activity and sport to aid physical, psychological, and social wellbeing. Originally developed to assist veterans to train for and participate in the Invictus Games, the program has extended its scope to promote recovery and wellbeing for all veterans through physical activity. There are two arms to the program, an exercise and performance program and a community adaptive sports program. Fundamental to the IPP is the provision of ongoing interdisciplinary allied health services (e.g., physiotherapy, podiatry, exercise physiology) to recovering military personnel and veterans. University students completing their placements within these allied health services work collaboratively with participating veterans to improve their wellbeing, providing one-on-one support and individually tailored programs.

A detailed explanation of the specific activities included in the individually tailored training is provided in the paper reporting the process evaluation of the IPP [13]. Briefly, once enrolled in the IPP, participants complete a high-performance testing session, performed by an Accredited Exercise Scientist (AES) member of IPP staff, with support from AES or Accredited Exercise Physiology (AEP) students. A testing protocol is developed for the participant, which may include activities such as maximal or submaximal testing, assessment of maximal oxygen uptake (VO2max), blood lactate, peak power (sprint or sustained), body composition, muscular strength, or isokinetic dynamometry. Participants repeat this testing protocol every three months to assess the impact of their participation in IPP.

A student trainer is assigned to each participant and works with that participant for the duration of the student’s 12-month placement. Participants are assigned a second student for the duration of their second year in IPP. One-on-one exercise training, using exercise equipment such as hydraulic weight machines, cardio equipment, and free weights, is provided by the student to the participant at UniSA’s exercise physiology clinic and gymnasium facilities, with the regularity of session delivery dependent on the needs of the participant and the student’s availability. Student trainers develop an individualised training program for the participant under the supervision of an AES/AEP member of the IPP staff. Training programs are reviewed every six weeks by the student trainer in collaboration with IPP staff. The student trainer then schedules regular training sessions with the participant, with sessions supervised by IPP staff.

Analysis of the IPP will be longitudinal, with the impact of engagement in the program on physical, psychological, social, and emotional factors to be assessed six-monthly across the duration of a participant’s involvement. This paper describes the expectations and experiences of the IPP from the perspective of a sub-set of program participants.

## Methods

### Conceptual framework

This research applied a pragmatist approach, which emphasises communication and shared meaning, leading to the development of practical solutions to problems [14]. Pragmatism does not align with a single philosophical approach nor reality, enabling multiple methods, worldviews and assumptions [15]; reflected in the use of quantitative and qualitative methods and analysis in this study.

### Participants

Participants in this study are a sub-set of participants involved in the IPP. We chose to include only the data of IPP participants who were yet to have participated in competitive activities such as the Invictus Games or Warrior Games. The rationale for this approach was to avoid the potential influence of involvement in these activities on participants’ perceptions of their experiences in the IPP.

### Quantitative data collection

IPP assessment and survey forms were obtained through data linkage following written informed consent from participants. Information collected from all participants in the IPP at baseline included background detail on the participants (age, marital status, education level, military service) as well as the completion of standard mental health and wellbeing surveys. These included scales used by the Australian Defence Force to assess mental health (Kessler Psychological Distress Scale, K-10) [16]; Posttraumatic Stress Disorder Checklist (PCL-C) [17, 18]; as well as the Alcohol Use Disorders Identification Test (AUDIT) [19], and the brief version of the World Health Organisation’s Quality of Life Scale (WHOQO- BREF) [20] and the Veteran Rand-36 (VR-36) [21].

#### K-10

The K-10, a ten-item inventory to assess generalised psychological distress, has good psychometric properties within civilian and military populations with a reported Cronbach alpha of 0.88 [22]. Responses to items are rated on a 5-point scale, from ‘not at all’ (1) to ‘all of the time’ (5), with scale scores ranging from 10 to 50. For the general population, scoring bands are low (10–15); moderate (16–21); high (22–29); and very high (30–50) [23].

#### PCL-C

The PCL is a standardised psychometric tool used to measure post-traumatic stress symptoms. The PCL consists of 17 questions which are scored from 1 to 5, with scores ranging from 17 to 85. It has excellent test-retest reliability (0.96) and good internal reliability (>0.82) [24]. The PTSD screening cut-off for military populations is 29 while the epidemiological cut-off for the same population is 53 [18].

#### AUDIT

The AUDIT is a screening tool to assess alcohol consumption and risky patterns of drinking [19]. Questions are answered in terms of standard drinks consumed, with a chart of standard drinks for different types of drinks provided for reference. The tool consists of ten questions, with the first eight being scored on a continuous 0–4 scale and the last two using a three-item scale (0, 2 and 4). The total score is obtained by adding all scores, with a range of 0 to 40. The cut-off score for this population is 8 [18]. Although this score is effective in detecting all cases of alcohol disorder, it has a low predictive value. Scores of 20 and over are more likely to indicate impairment and the need for referral for treatment [18].

#### WHOQOL-BREF

The WHOQOL-BREF is a shortened, 26-item version of the original 100 item WHOQOL [20], which assesses overall quality of life, general health and four life domains: physical health, psychological health, social relationships, and environment. Correlations between the four life domains for the 100 item and BREF are good (0.89 to 0.95), with acceptable to good internal consistency for the domains (Cronbach alpha 0.66 to 0.84) [20]. Scores from the WHOQOL-BREF can be transformed into percentages based on the specific population scores for each domain, which has been applied here to allow for comparison with other data sets. Community based population data indicate a score above 70 as the norm across the domains [25].

#### VR-36

The short form-36 is a widely used quality of life measure assessing eight health concepts: physical functioning, bodily pain, role limitations due to physical health problems, role limitations due to personal or emotional problems, emotional well-being, social functioning, energy/vitality, and general health perceptions [26]. These eight health concepts are often reported as a physical component summary (PCS) and a mental component summary (MCS) with scores from 0 to 100, where 100 denotes the best health [27]. The scale also includes a single item that provides an indication of perceived change in health. The VR-36 is a veteran specific version of the scale with acceptable to good internal consistency (Cronbach alpha: 0.66 to 0.89) [21].

### Semi-structured interviews

Semi-structured interviews were conducted in person [SF]by a female PhD Candidate experienced in qualitative data collection at [location removed for peer review] and audio recorded. An interview guide was developed (see S1 File) and piloted among the research team. Interviews were transcribed verbatim and lasted between 12 and 73 minutes, with an average length of 37 minutes.

### Ethical considerations

Prior to each interview, participants were briefed about the aims of the research and provided with the Participant Information and Consent Form (PICF). Written informed consent was obtained. Participants received a copy of the PICF and a copy of the Guidelines for Volunteers to keep. Every effort was made to minimise potential discomfort to the participants. Interviews were conducted next to the university’s health service, so that immediate access to a General Practitioner could be provided in the event that a participant required additional support. Electronic data were stored on a password protected university network drive and accessible only by the researchers.

### Ethics approvals

This research was approved by the Department of Defence and Veterans’ Affairs Human Research Ethics Committee (DDVA HREC, protocol number 039–18) and the University of South Australia’s Human Research Ethics Committee (HREC, protocol number 201362).

### Rigour and trustworthiness

Reflexivity, the process of examining one’s own role in shaping the study, was employed by all members of the research team as they undertook interview data collection and analysis of the interviews. A conscious effort was made to examine and explicitly acknowledge any aspects of personal background, culture, and experiences which might shape the study and the direction in which it was progressing [28]. Further, prolonged engagement also occurred as interviews were undertaken over the space of three years, a strategy thought to help establish trust with participants and yield thicker, richer qualitative data [29]. The researcher who undertook the interviews (SF) spent extensive time with participants across the duration of this work, attending IPP sessions and program events in addition to research activities. Through this engagement, the researcher established relationships with the participants and came to understand their experiences as a veteran living with physical and/or psychological conditions, and the impact that these conditions had on their capacity to engage not only in the IPP, but also in day-to-day life. Other researchers in this team have worked with this population, and the veteran population more broadly, for more than five years, with an understanding of the impacts of service on veterans and the veteran family unit. Beyond this, some of the researchers have extensive experience in the sports psychology and physical activity spaces. This combined experience enabled the data to be viewed through multiple perspectives when it came to discussion and refinement of the themes.

### Data analysis

Survey data were entered into data management files, checked for accuracy and completeness, and are reported as means and standard deviations. Interview data were analysed using thematic analysis [30]. Following transcription, three random interviews were checked for transcription accuracy by a second researcher. Interviews were coded by one researcher (SF) and themes were collated. A second researcher (DP) independently coded the interviews. The use of multiple coders was intended to increase the rigour and trustworthiness of the analysis, not for the purpose of achieving consensus but to instead apply more than a single perspective to the narratives identified. Coding and themes were developed through a mixture of inductive and deductive approaches to analysis. Initial themes related to previous life experience, expectations of participation, and outcomes of participation were preconceived, but the analysis provided scope for an inductive approach to formulate additional themes. Qualitative data analysis also involved peer debriefing with the research team throughout the data collection and data analysis process. As mentioned, the experience of the research team proved beneficial in identifying narratives with the data and refining themes.

## Results

### Population descriptives

Fifteen participants (n = 13 males, n = 2 females) consented to the release of their IPP data. The majority (n = 11) were between the ages of 40–49 years (2 were 30–39, and 2 were 50–59), 11 were married or had a de facto partner (1 was never married and 4 were divorced), 13 were medically discharged (2 declined to answer), and 7 were in full-time work with 5 unable to work, and 1 retired. All three services were represented: Airforce (n = 4), Navy (n = 6), Army (n = 4), with one participant not responding to the question. Participants had varied formal education: 1 had a master’s degree, 3 had a post-graduate diploma, 1 had a bachelor degree, 3 had vocational training (outside of their service), 3 graduated Year 12, and 4 left school before completing Year 12.

### Quantitative results

The mean scores and standard deviations for the surveys completed by the participants at baseline are provided in Table 1. One participant declined to complete the surveys, and one other only completed the K10, AUDIT and WHOQOL-BREF.

**Table 1:** Psychological distress, wellbeing, and quality of life data.

	n	Mean	SD
K10 score	14	24.00	11.04
PCL-C score	13	40.46	19.58
AUDIT score	14	3.86	3.63
VR-36 Physical Component Summary	13	49.53	30.75
VR-36 Mental Component Summary	13	52.58	34.83
WHOQOL-BREF Physical score	14	58.48	19.62
WHOQOL-BREF Psychological score	14	60.71	20.18
WHOQOL-BREF Social relations score	14	57.14	20.87
WHOQOL-BREF Environment score	14	64.64	16.40

Five of the participants had very high K10 scores (>30), and scores above the norm for PCL-C, whilst one would be classified with an alcohol disorder (scores over 8), with no participants scoring above 20 (indicative of requiring referral). Similarly, five had PCS and MCS (composite scores from the VR-36) that were below population norms [31]. However, while the averages for the WHOQOL-BREF were below population norms, four were above the norm for all four life domains [25].

### Qualitative findings

Interviews were undertaken with all 15 veterans, between August 2018 and April 2020. None had previously participated in either the Invictus Games or the Warrior Games. Findings from the interviews indicated that few veterans had expectations upon joining the IPP and had mostly positive experiences as a result of participating in the IPP. The key themes identified through analysis included: **Life experiences prior to participation in the IPP** (previous involvement in physical activity and sport, physical and psychological wellbeing, transition from Defence and engagement in society), **Making a choice to participate in the IPP** (the right time to join the IPP, hesitation and drivers), **Expectations of participation in the IPP** (unsure of what to expect or no expectations, improved fitness, social engagement, participation in Invictus Games), **Impact of participation in the IPP** (physical impacts, psychological impacts, social impacts, health literacy and self-awareness, belonging and feeling supported, giving back), and **Future Plans** (improved fitness and recovery, competitive opportunities, opportunities for physical activity and social engagement, setting goals).

### Life experiences prior to participation in the IPP

In order to be able to fully explore veterans’ perceptions of the impact of their participation in the IPP, it is important to firstly understand what their life was like before they joined the program, and how these prior experiences may have influenced their reluctance or enthusiasm to be involved in IPP, their expectations about IPP and its potential impact on their lives. This includes understanding veterans’ prior engagement in sport, their mental and psychological wellbeing, and their experiences prior to joining the IPP.

### Previous involvement in physical activity and sport

While all participants reported being physically active when they were younger, and some at state and national representative levels, most stated that their physical activity had greatly decreased or had ceased completely due to physical and/or mental health issues, the majority of which were associated with their service. Despite many military service roles being physical in nature, engagement in physical activity as part of their service was not raised by participants, although this specific aspect was not necessarily viewed as important by the researchers with respect to it being a driver of participation in the IPP. Veterans who were engaged in physical activity immediately prior to joining IPP reported going to the gym, swimming, jogging, running half-marathons, and social cycling, with one stating that doing so was ‘more for mental health’ reasons (P3). A few of the participants acknowledged the link between them being physically active and the role this played in their mental wellbeing:

I knew that sport made me feel better about myself . . .I just know that the sport and training helps me to focus away from that [distractive thought pattern] and, in a sense, channel my focus . . .towards just, it’s building myself up. (P5)

My PTSD goes back to 2005 . . .and so I’m a master avoider of things, and the way that I’ve been able to suppress and avoid it is through my physical activity. By setting goals, pushing myself too hard, which probably contributed to the downfall, but the physical aspect of things is a huge part of keeping me stable. (P15)

### Prior physical and psychological wellbeing

Participants detailed a range of health issues and negative physical and psychological experiences prior to joining the IPP. All participants lived with physical and/or mental health issues. Participants stated that they were ‘isolated’, ‘just in survival mode, each and every day, trying to push through’ (P2); or felt ‘lethargic’, ‘miserable’ and not motivated to ‘get up and do anything really’ (P7). For others, there were reports of previous suicidal ideation and self-harm:

I was extremely suicidal. And that was a horrible place to be in. (P6)

I’ve had a relapse of depression, major depression. I’ve had thoughts of killing myself and things like that. I have anxiety. (P8)

. . .my mental health is a little bit different. I do have a lot of ‘poor me’ days, and I really struggle. (P13)

### Transition from defence and engagement in society

Multiple participants spoke about negative experiences while, or just after, they transitioned out of the Defence Force. Some explained how they felt that their career was ‘cut short’ due to health issues and being medically discharged, with no support offered. For others, their health deteriorated further after discharge:

When they offered me a medical discharge, I just took it not knowing what was gonna happen. Back then things were very different. I was walked to the main gate of [location] and I was given a cheque for $2000 and wished good luck. And that was it. There was no rehabilitation. There was no assistance with getting another job or anything like that. It was just out on the street. (P2)

[I] . . .discharged in 2007, probably not in a very good mental health state and certainly not in a good physical state either. My physical health deteriorated quite a lot over probably a six or a seven-year period and of course my mental health as well. (P3)

Some veterans spoke of their struggle to fit into society post-transition from Defence. For some, this was attributed to the loss of structure that came with the military lifestyle, while for others, there was a sense of being on a ‘different planet’ to the people they were socialising with:

. . .it was very difficult getting out of the Defence Force after being in for so long because you become indoctrinated and you know what’s gonna happen, when it’s gonna happen, where it’s gonna happen, where you have to be. Everything is structured. And to go from that through a door that you have no control over anything anymore. You don’t know what’s coming up. (P1)

But socially I’d try to fit into groups myself and they were just on, different concerns, on a different planet, so to speak. It’s them that’s on different planet, not me. So they were all more worried about, you know, what they were gonna wear and their hair and all these things and, you know, what they’d look like to other people whereas me, it’s very different. (P6)

I find that I have a lot of trouble still in civilian life, talking to people at sporting clubs and that sort of thing, because I’ve got a very different mindset and structure to the way I live life. (P14)

### Making a choice to participate in the IPP

**The right time to join the IPP?** Perceptions about when it was the right time to join the IPP and some of the factors associated with making a choice to participate were varied among the veterans interviewed. Many thought that this type of program was ideal for anyone transitioning out of Defence, particularly those with physical and mental injuries; however, the need to engage people in the program early, for example before they left Defence or before their health deteriorated further, was raised. For others, there was a perception that veterans need to be motivated to get involved:

. . .when someone is introduced to the program, it’s because either someone has pushed, directed them in that direction or that pathway, as myself telling another veteran to go and do this because what it’s done for me . . .[or] someone’s gone to [IPP staff member] and saying: “Can you help this person?”. (P4)

You’re going to get the person that’s going to walk straight up and say, ’I’d like to do this program.’ And you’re also going to get probably some–what happened to me, like you need someone to just go, ’Look, here. Sign this. Do this.’ And then because I can only talk army, generally once a military person is committed to something they will do it. Once they’re in, they’ll turn up. (P13)

#### Hesitation and drivers

Some participants spoke of lacking confidence when it came to joining the IPP, partly attributed to a fear of failure or not meeting people’s expectations of them. For others, hesitation was driven by a resistance to change or uncertainty and unease about what the program was about:

… physical fitness and wellness is a long-term objective that has been out of my life for more than 15 years and I’m frightened of failure. (P6)

Others spoke about wanting to be involved in something as a driver of their participation or feeling like they had exhausted opportunities to support their wellbeing and that being in the IPP was worth trying:

Look, it’s taken me a lot to come out of my shell and I think it depends on how you are mentally focused, if you are or not. So, for me it was like I have to get it; I have to do something; I have to get involved with something. (P10)

I was at a point where I thought there was nothing I could do, so I thought what the hell, I’ll give it [IPP] a try. (P7)

### Expectations of participation in the IPP

#### Unsure of what to expect or no expectations

Most veterans reported being unsure of what to expect or that they did not have any expectations about their involvement in the IPP. In part, this was attributed to a perception of lack of communication about the IPP’s purpose and components. For others, expectations were intentionally kept low in order to avoid disappointment, and for one veteran, the program was simply just something to do:

Look, before I joined, I didn’t really have any expectations because I’d been in a place where, you know, I just really wasn’t really feeling much at all. So, when I came to the program I went ‘you know what, I’m gonna go into it with a clear head. If it works, it works. If not, at least I’ve tried something’. (P7)

So, I think having no expectations was best way to approach it because… you’re not gonna be disappointed and you’ve got everything to gain (P1).

I really came in with zero expectations. It was for me personally, it’s just something to do at the moment. And I know that sounds really horrible, but it’s something to do. (P13)

#### Improved fitness, social engagement, participation in Invictus Games

For those who did have expectations, most were related to enhancing their recovery, improving fitness, and losing weight, with others hoping to increase their social engagement or take the opportunity to be part of a team again. Selection for the team to represent Australia at the Invictus Games, as a result of improved fitness, also appeared to be an expectation of participation in the IPP:

My expectations were to hopefully improve my physical condition . . .and improve my mental illness as well. So, if I really improved my activity and mobility and my body wouldn’t be as sore and then I’d be a bit happier. (P9)

Well, my expectations are that I will increase my level of physical fitness which will take some of the stress off my body and allow me take on more mentally taxing tasks.

And as a side-effect of increasing my physical fitness, I hope I will lose a little bit of weight and therefore improve my self-image. (P11)

. . .I hoped I was actually in a Program where I was doing things physically all the time and actually improving my fitness would give me a better chance of selection [for the Invictus Games].’ (P3)

### Impact of the IPP on veterans’ wellbeing

According to all IPP participants who were interviewed, taking part in the program had a positive impact on their wellbeing. Some spoke about the positive outcome in general terms, while others discussed its physical, psychological, and social impact, including how these three aspects interacted. Veterans perceived that taking part in the IPP improved multiple aspects of their lives, and for some, there was an influence on family too:

Probably, I would say probably since getting really hooked into the Pathways Program, I think it (life) has probably improved significantly… I’ve got so much out of the program . . .I can’t really begin to say how much I got out of the Program. (P3)

I feel stronger and fitter. I feel healthier. Yeah, I feel like I want to help myself. I feel that’s one of the main things. Because it’s making me ‐ it’s encouraging me to help myself . . . I was depressed, frustrated, social anxiety as well. And now I come out and I talk to people and it’s pretty good actually. It’s been so good for me. (P8)

I went from being a survivor to starting living again which was really, really good. (P2)

We are doing more outside that we wouldn’t have previously, prior to the program. For instance, we quite often go for bike rides out through [place], as a family, so that’s helped us get out and about. That’s something we wouldn’t have done prior to the program. (P12)

#### Physical impact

Taking part in the IPP was reported to have a large impact on veterans’ physical wellbeing. For some veterans, this impact included learning new skills that enabled improvements to their fitness and strength and being able to get more out of their body, although some acknowledged that they were not at the level they had been previously:

. . .when I’m doing that sport, I can focus on what I’d been taught . . .it’s amazing when you do something by yourself and you haven’t been taught, and you get it to a certain level and then you, you’re brought up to a new level by instruction, how much more you can get out of it and how much more you can continually surprise yourself just through that whole repetition and building on it… (P5)

There’s certainly been an improvement to, you know, a year ago to now on, you know, my ability to be able to do the sports and stuff like that, but like I said, I’m still not quite back to where I was, but, you know, I’m certainly trending towards that direction, I guess. (P3)

Further physical impacts of participation noted by veterans included weight loss, increased muscle mass, and in some cases these physical changes were associated with perceptions of a reduction in pain. Most participants commented on becoming fitter, faster, and stronger due to taking part in the IPP. They also appreciated being able to get a personalised and tailored exercise program based on their existing injuries and health conditions:

I started the Invictus Pathways Program and started doing the exercises and started doing the riding, I actually gained 4 kilos of muscle mass and lost 4 kilos of body fat. We even, when they measured the fat around your body parts, they measured that I reduced in everything, so that was really quite good. (P2)

I still have knee pain, however due to losing weight through this program, the knee pain is not as bad as it used to be . . .And just the knowledge that this was all being written down so that they can tailor a program to my specific needs not to a generic person’s needs. (P7)

. . .for me personally, it’s had better results in the last 12 months [in IPP] than it has five years of doing rehabilitation through the military . . . I’m healthier. It’s amazing. My injuries haven’t got worse. I don’t think I’ve been to the doctor in the last two years for pain medication whereas I used to be on heavy stuff, like Tramadol, Endo and all those painkillers. (P4)

#### Psychological impact

A majority of participants described experiencing improvement in their mental health due to taking part in the IPP, while acknowledging that IPP was not a ‘cure-all’. Veterans highlighted that while they still struggled at times, they either felt more positive overall, or that training in the IPP helped them manage when they were feeling down:

. . .yeah, it’s just, it’s helped so much. You know . . .I still have crappy days like everyone does, but it’s definitely, it’s not like a week of feeling down . . .it could be like a couple of hours or a day at most where I might feel like I’m sliding into a hole and then, you know, the next day I’m back at the gym and just releasing all that negativity and, you know, set myself more goals to just keep going. (P7)

I think definitely being part of something structured, like the Pathways Program, where I actually look forward to going, not just for the fitness side or my just wellbeing, but for my mental health . . .(P3)

I still have nightmares. I drink shit loads of alcohol just to sleep at the night-time. I probably take too many drugs to sleep at the night-time. I find myself waking up lathered in sweat. But having started this Program, it’s allowed me to force myself to come back in to a… I call it the edge of society. (P1)

Improvements in veterans’ confidence, their general outlook, mindset, and motivation were also reported, with veterans discussing having goals and something to focus on when they feel like they were struggling:

Oh, it’s probably made me a bit more confident again . . .I discharged from [service] . . .I wasn’t in a very positive frame of mind at all. I was probably quite frustrated, disappointed with how I discharged. My career was cut, you know, shorter than what I would have liked . . .so it has given me a chance to rebuild that confidence…(P3)

And I loved it from the very first session, you know. I felt a change in me, in my mindset I guess, from the very first time I walked into the gym with my trainer. (P7)

Even though now I’m struggling at the moment, I know that in a few months’ time I will be back up in that happy place. Cause if I didn’t have that, I wouldn’t know what I would be doing in three months’ time. So I’ve got goals for a change. I didn’t have those before. (P2)

#### Social impact

For all veterans who were interviewed, getting involved in the IPP increased their social engagement. Many stated that they had been completely on their own and had avoided any type of socialisation, until they joined the IPP. Being part of the program provided them with “mateship” (P7), and being part of a team again, which they found very beneficial. This extended to being and feeling part of a community again:

From being on my own for so long and being in a dark place for a long time, it was nice to step forward and sort of become part of a team, you know. It’s like when you’re in the military, you not only rely on other people to help, but you’re helping other people. So it’s all, it’s a family. It’s a community. It’s the social side of it . . . (P2)

. . .that actual feeling of community, being part of something again. I think for me that’s been the most positive. I mean obviously, doing the sport and all that sort of stuff is fantastic, but to you know, it wouldn’t be as nice if you come here and no one spoke to you or, you know, you came in and just did your gym work and get out sort of stuff. I mean you don’t feel that way here. (P3)

. . .I probably didn’t think that, from a relationship or friendship [aspect], the non-physical aspect of the program would be as good as it turned out to be and how much it supported me. (P15)

I think the socialisation and integration are the most important bits. Because generally most military people will do the physical stuff . . . you’re creating an environment where there’s a big bunch of people, you’re getting people out. (P13)

#### Health literacy and self-awareness

Beyond the physical, psychological, and social impacts, some veterans reported feeling that they had also learned a lot about exercise, injuries, and the need for recovery as a result of participating in the IPP. Beyond this, one veteran described how they also felt that they learned a lot about themselves:

. . .if I walked away from here, I’d feel like I’ve learnt so much. (P4)

I think this first part of the journey, I’ve really kind of worked out what, I’ve learned a lot about myself. (P3)

#### Belonging and feeling supported

The role of IPP staff and students was suggested by veterans to be vital to their success in the program and veterans spoke of a feeling of being respected. Veterans commented on staff and students’ dedication to the program, that they appreciated them attending sporting events for veterans to offer support, even though this is not part of their work and study requirements:

. . .we had a whole team of volunteers out there [at event] . . .looking after us and that was just incredible. I mean, the morning after the event, we were all standing around, talking and thanking everybody that was there and a lot of us veterans were, you know, almost in tears because we couldn’t believe the amount of support we were getting from other people. It was really nice. (P2)

They [staff and students] showed us the respect we hadn’t been shown by other people. And we work with them. We’re working together with the staff here at UniSA. That was really impressive. Like I said, I was blown away by the way we were looked after and we have, we still continue to be looked after. (P4)

And this might sound condescending, but I don’t think the kids–yeah, they are for me–I don’t think they actually quite get it. I think they understand these guys have got mental health stuff and you’re going to learn a bit, but I don’t actually think how much they give to us–I get how they understand what we’ll give to them. With the training and the chats and everything. But I don’t think they understand what they give to us. (P13)

#### Giving back

Veterans reported that they appreciated the opportunity to give back to the students by supporting their education, through participation in the practical aspects of the IPP and by providing feedback related to the skill development of students. Others offered to champion the IPP or contribute their own experience, skills, and knowledge to the program:

And to see some of them coming out of their shells, it’s a win-win situation. So, I’ve gained a lot out of this through seeing them develop… it’s like what you guys give to me, I wanna give back . . .It’s our duty to give back to those who have given to us. (P4)

Like I told them, I said it’s a learning thing for both of us . . .I really want them to learn . . .by all means, I’m happy to be their test dummy. It benefits them for their future as well. And that’s sort of why I joined up with the program, is to really help those guys and girls out and get as much out of it. (P10)

### Future plans

As one of the main aims of the IPP is rehabilitation and reintegration, including supporting veterans to get involved in sport and join clubs, it is useful to understand veterans’ plans for their immediate future in the IPP, and ultimately, when they transition out of the IPP.

#### Improved fitness and recovery

Veterans described how they intended to use their time in the program to improve their fitness and to continue with physical activity generally. For those with injuries, their focus was on recovery first and then a return to training in the IPP:

And that’s the goal now, just to recover from the injury . . .[the] type of injury that I’ve got now is one that’s very difficult to recover from, so I just gotta get my head around it and slow right down and take one day at a time. (P2)

#### Competitive opportunities

Other veterans spoke about the opportunity to be involved in either the Invictus Games, Masters Games, or the Warrior Games, the need to be adept at multiple sports to increase their chances of selection, and how this influenced their approach to ongoing involvement in the IPP:

I’d love to be selected in the team and go overseas and represent; absolutely definitely. (P11)

This is it; I kept my eye on Invictus trials. If I’m going to go to Invictus trials and I’m mad keen to get to Invictus trials I have to get back in the pool ‘cause I’m going. (P13)

Going to Hague and we’ll have a team of maybe 35, so I’m gonna need to be able to do multiple sports to get selected for that. (P7)

#### Opportunities for continued physical activity and social engagement

For veterans who had already become involved in sports and organisations external to the IPP, there were plans to continue with that engagement. For others, there were longer term plans associated with getting involved in new physical activity opportunities including cycling, wheelchair basketball, wheelchair rugby, athletics, triathlon, and rowing. Some veterans enjoyed competing, while others were more interested in the social aspects of team sports. For some, the potential for long-term impact was also mentioned:

. . .so in summer I’m going to make myself known to the [name] Athletics Club and try and become a Master [level of athlete]. See how we go with that. [P13]

. . .you know, if I happen to be one of those 60-year-old fit blokes on a bike, then I’m really happy to be that. (P5)

#### Setting goals

For some participants, their involvement in IPP had assisted them in setting goals for their future that extended beyond the program, to include marching in an ANZAC Day parade, and a return to study:

My life goals are to be able to march in next year’s ANZAC Day Parade. I wasn’t able to march in this year’s ANZAC Day Parade because I don’t have the stamina to march three kilometres, so I had to sit in a wheelchair while somebody . . .pushed me along. (P11)

My goal, actually, the year after, is to study at UniSA in the health sciences area. And that’s purely because of my engagement with UniSA . . .And so just the relationships I’ve built at the uni makes me feel safe about going there, and that’s a huge thing. (P15)

## Discussion

The purpose of this study was to examine the perceptions of the veterans who participated in the IPP with respect to the impact of the IPP on their physical and psychological wellbeing. To assist contextualisation of these perceptions, we also sought to understand the life circumstances of veterans prior to commencing with IPP and their expectations of participation in the program.

The survey data collected prior to the interviews provided an overview of the population and indicated that the majority of veterans had high levels of psychological distress. This was reflected in the interviews, where veterans described the ongoing impact of their service on their physical and mental health. Some veterans described negative experiences transitioning from the military and into civilian society, with survey data supporting that for some participants, engaging in social relationships and occasions was difficult.

The qualitative findings suggest that the impact of the IPP on physical, psychological, and social factors was positive for all interviewed participants, irrespective of whether their survey responses indicated that they had low or very high levels of psychological distress. How this perceived impact translates to objective measures of psychological distress remains to be seen and is the subject of the ongoing evaluation of the IPP for subsequent cohorts of participants.

Interdisciplinary programs of this nature, that include allied health services such as physiotherapy, podiatry, occupational therapy, and exercise physiology, and specifically those that are student-led or that involve students in physical training or reablement, do not exist in Australia nor internationally. Two other Australian universities offer a mono-disciplinary (exercise physiology) supported program (based upon IPP) to veterans and student veterans, and information about the impact associated with participation in these programs is yet to be published. Irrespective of this, there is growing evidence for the benefit of exercise for PTSD and other mental health conditions generally in the veteran community. Beyond the potential for positive impact on mental health, involvement in physical activity has been shown to increase social engagement. Additionally, social relationships and particularly the influence of family, have been demonstrated to be key for initiation of engagement in adapted physical activity for people living with disability [32].

For the veterans who participated in the current study, there were perceptions that programs such as the IPP would be ideal for anyone who had been injured during service or was transitioning out of Defence, and further, that this particular program should be introduced prior to veterans leaving the Defence force. The timing of engagement agrees in part with other work that suggests transitioning members should be notified of the availability of a support program during the period between their notification of release from service and their actual release [33]. The same study identified that for veterans who had transitioned from service, four-to-five years post-transition was the ideal time to engage in a program of this type [33].

Beyond concepts related to the most appropriate time to join this type of program, some veterans in the current study perceived that veterans need to have a sense of self- motivation to be involved in a program of this nature, irrespective of the point at which they engaged in a program. One veteran mentioned that due to the ‘type’ of people in the military, once they committed to something, they ‘stuck it out’. Research related to predictors of engagement of veterans in ‘bespoke recovery pathways’ found that ‘feelings of failure or inadequacy’, associated with self-perceived competence, was a predictor of engagement, as was perceived social support [34]. Further, social support was associated with long-term engagement in recovery pathways. Such concepts are relevant to the IPP, particularly with respect to the duration of the program and the opportunities to build social support through engagement with students and other IPP participants, with shared experience, during this time. It is here too that the community adaptive arm of the program can play a role, with the intention that there is direct support for transition to engagement with the community and community-based activities. These concepts, and if the program has successfully supported its participants, should be further examined as the program continues.

The positive impact of physical activity for participants and engagement in the social aspects of the program were identified as being beneficial for the veterans’ psychological wellbeing. For some, a sense of ‘belonging’ was articulated as a positive impact of participation in IPP. In some respects, the IPP environment may reflect that of the military environment which can contribute to a sense of familiarity, belonging or being part of ‘something’ again [8]. Belonging has been established as a component of what is considered ‘quality’ participation in physical activity [35].

Being able to ‘give back’ to the students and the program, by contributing to the students’ learning, was identified as a driver of participation in IPP by some of the veterans interviewed. The perception of service and continuing to serve to ‘ . . .give back to the community, and to make a difference in the lives of others’ [36] has been identified in other research investigating the involvement of veterans in programs.

Veterans in this study perceived that they were fitter and stronger and for some, there was an increased sense of knowledge about physical activity and how their own physical composition was impacted by activity. Being physically trained by students, who have more time to spend with the veterans during their time at IPP than what might occur in standard healthcare settings, may lead to the sharing of more health-related information over that time. This in turn may contribute to increased knowledge for the participant. Similar concepts have been identified in peer-led programs involving veterans, whereby engagement in health-related programs that provide time and consider the veteran’s goals and health more holistically may lead to greater engagement of veterans in their own health and care [37].

In the current study, some of the veterans described seeking, or intending to seek, opportunities to engage with sporting organisations outside of the IPP; this is an intended outcome of participation in the IPP. Research examining the involvement of veterans in parasports identified a number of pathways, and phases within these pathways, that facilitated ongoing physical activity for veterans living with a disability [35]. These pathways include physical activity that stems from rehabilitation, invitation to participate or through watching others participate and succeed (vicarious experience), and through seeking new programs to be involved in [35]. Aspects of each of these pathways are embedded within the structure of the IPP and are reflected in the interviews with veterans. Further, while the objective of this paper was not to directly test theory, the structure and content of the IPP is founded on evidence-based practice. As such, underlying theories such as Bandura’s Social Cognitive theory [38], the theory of social connectedness [39], or biological mechanism explanation [40] are among those that could explain the impacts experienced by the participants in this study.

One pathway in the IPP is to support veterans to train for the Invictus Games. While none of the veterans interviewed had competed in the Invictus Games at the time of interview, members of the IPP qualified for the Australian team in 2018 and 2020. Recent research suggests that participation in the Invictus Games supports rehabilitation and physical activity engagement but calls for additional research [41]. The experiences of IPP participants who qualified for and participated in the Invictus Games will also be examined as part of the ongoing evaluation of IPP.

## Limitations

There are limitations with this study, some of which relate to program-specific factors. In its initial format, the IPP was not intended to be a holistic approach to wellbeing, instead focusing on physical activity and engagement, and where desired, a pathway for participation in the Invictus Games. Frameworks that incorporated the ongoing collection of data about factors related to mental wellbeing and other behavioural factors were not in place and as a result, we are unable to report objective mental wellbeing outcomes for this population. This does, however, indicate the need for the development and implementation of process, impact, and outcome evaluation frameworks that will support the ongoing activities of IPP.

## Conclusion and future directions

The Invictus Pathways Program has been shown to have a positive impact on the physical and psychological wellbeing of the veterans who participated in its initial stages. Impacts have extended to re-engagement in community adaptive sports, as well as providing veterans with a sense of belonging and the capacity to continue to serve the community through supporting student learning. As the program evolves, the longitudinal impact of participation, for veterans and their families, will be assessed.

## Supporting information

S1 File Interview guide. (PDF)

S2 File Audit trail example. (PDF)

S3 File COREQ checklist. (PDF)

S1 Dataset Data reported in Table 1. (XLSX)
