# Supplementary material for: The physical, psychological, and social impacts of participation in the Invictus Pathways Program: A qualitative analysis of veterans’ perceptions and experiences
Source: PLoS One. 2023 Oct 30;18(10):e0287228. doi: 10.1371/journal.pone.0287228 (PMC10615282; doi:10.1371/journal.pone.0287228)
Supplement: S1 File — (PDF) [file pone.0287228.s001.pdf]

Interview Guide for Invictus Pathways Program Participants

1. Please tell me a little about yourself.
2. Have you been involved in any sports and recreation prior to joining the Invictus Pathways Program? (*Prompts: type of sport, length of engagement, frequency, duration, level of achievement/success*)
3. What was your life like prior to starting the Invictus Pathways Program?
4. What were your expectations in regards to the Invictus Pathways Program?
5. How did you expect that taking part in the Invictus Pathway Program would affect you? (*Prompts: from physical, social and psychological perspective*)
6. How do you think that being in the Invictus Pathways Program has impacted your life? *(Prompts: from physical, social and psychological perspective*)
7. In your opinion, which aspects of the program are most beneficial? Why do you think these aspects are most beneficial?
8. In your opinion, which aspects of the program could be improved? Why do you think these aspects can be improved? How do you think these aspects can be improved?
9. Do you have any further comments you would like to make in relation to what we have talked about?
