# Supplementary material for: The physical, psychological, and social impacts of participation in the Invictus Pathways Program: A qualitative analysis of veterans’ perceptions and experiences
Source: PLoS One. 2023 Oct 30;18(10):e0287228. doi: 10.1371/journal.pone.0287228 (PMC10615282; doi:10.1371/journal.pone.0287228)
Supplement: S2 File — (PDF) [file pone.0287228.s002.pdf]

S2 File Audit trail data analysis example

Example of analysis for the theme: Impact of the IPP on veterans' wellbeing (not all sub-themes included)

Theme	Impact of the IPP on veterans' wellbeing					
Sub-themes	Physical Impact		Psychological Impact		Belonging and feeling supported	
Categories	Improvements to fitness and strength	Perceptions of reduction in pain	Improvements in confidence, outlook, mindset, and motivation	Still struggled at times, but IPP helped	Being respected	Staff and students' dedication
Codes (examples)	Been an improvement in ability		More confident		Showed us respect	A whole team of volunteers out there
	Fitter, faster, stronger		Struggling at times			Support from other people
	Pain is not as bad as it used to be		Got goals now – didn't have before			
Quotations	'I feel stronger and fitter. I feel healthier.'		It's probably made me a bit more confident again		'They [staff and students] showed us the respect we hadn't been shown by other people.'	'...we had a whole team of volunteers out there [at event]...looking after us and that was just incredible.'
	'I still have knee pain, however due to losing weight through this program, the knee pain is not as bad as it used to be…'		'…I'm struggling at the moment, I know that in a few months' time I will be back up in that happy place. Cause if I didn't have that, I wouldn't know what I would be doing in three months' time. So I've got goals for a change. I didn't have those before.'			'…a lot of us veterans were, you know, almost in tears because we couldn't believe the amount of support we were getting from other people.'
